# Transcription Factors in Eosinophil Development and As Therapeutic Targets

**DOI:** 10.3389/fmed.2017.00115

**Published:** 2017-07-24

**Authors:** Patricia C. Fulkerson

**Affiliations:** ^1^Division of Allergy and Immunology, Cincinnati Children’s Hospital Medical Center, Department of Pediatrics, University of Cincinnati College of Medicine, Cincinnati, OH, United States

**Keywords:** hematopoiesis, eosinophilopoiesis, transcriptional regulation, eosinophil development, eosinophil lineage commitment

## Abstract

Dynamic gene expression is a major regulatory mechanism that directs hematopoietic cell fate and differentiation, including eosinophil lineage commitment and eosinophil differentiation. Though GATA-1 is well established as a critical transcription factor (TF) for eosinophil development, delineating the transcriptional networks that regulate eosinophil development at homeostasis and in inflammatory states is not complete. Yet, recent advances in molecular experimental tools using purified eosinophil developmental stages have led to identifying new regulators of gene expression during eosinophil development. Herein, recent studies that have provided new insight into the mechanisms of gene regulation during eosinophil lineage commitment and eosinophil differentiation are reviewed. A model is described wherein distinct classes of TFs work together *via* collaborative and hierarchical interactions to direct eosinophil development. In addition, the therapeutic potential for targeting TFs to regulate eosinophil production is discussed. Understanding how specific signals direct distinct patterns of gene expression required for the specialized functions of eosinophils will likely lead to new targets for therapeutic intervention.

## Introduction

Eosinophils differentiate in the bone marrow from an eosinophil lineage-committed progenitor (EoP) that is derived from the granulocyte/macrophage progenitor (GMP) in mice and the common myeloid progenitor or an upstream multipotent progenitor in humans (1, 2). Cell fate choices, including lineage commitment, are specified by the action of primary, or lineage-determining, transcription factors (TFs) and then reinforced by induction of secondary TFs that orchestrate gene expression and lineage commitment and differentiation. TF concentrations can be important, as lineage-determining TFs can antagonize each other’s activity (3, 4). We have recently shown that markedly more transcriptome changes (1,199 genes) are associated with eosinophil maturation from the EoP than with eosinophil lineage commitment (EoP from GMP, 490 genes), highlighting the greater transcriptional investment necessary for terminal differentiation (5). These dynamic changes in gene expression during eosinophil development included a repertoire of TFs, many of which had never previously been associated with eosinophil development (5). New information from genome-wide and single-cell RNA sequencing (scRNA-seq) studies have built upon well-established models of transcriptional regulation of eosinophilopoiesis. The molecular regulatory network that yields functional, mature eosinophils from EoPs is slowly being delineated. Defining how eosinophil production is regulated is critical to understanding how dysfunction of the immune response results in eosinophil overproduction and will likely lead to new eosinophil-targeting therapeutics.

## Eosinophil Lineage Commitment

**Figure 1:**
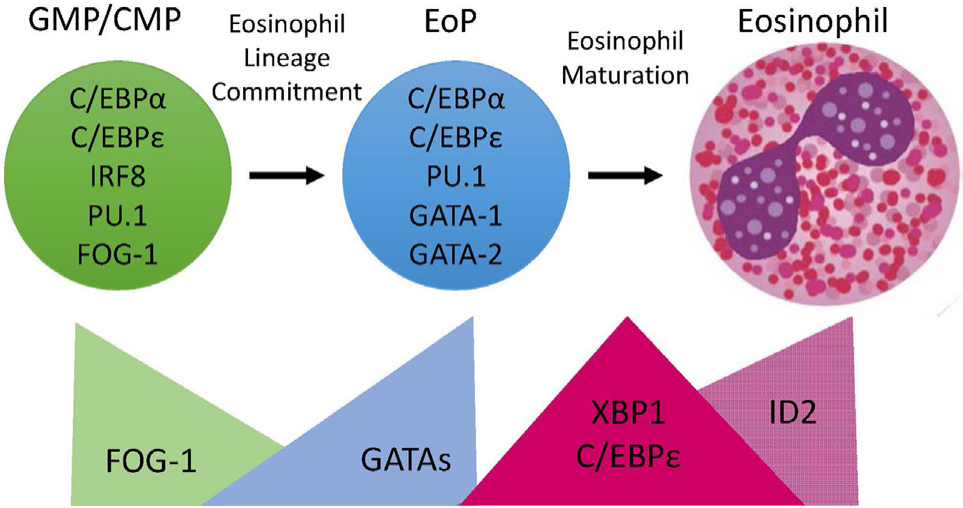
Transcription Factor (TF) expression during eosinophil development. Eosinophils differentiate in the bone marrow from an eosinophil lineage-committed progenitor (EoP) that is derived from the granulocyte/macrophage progenitor (GMP) in mice and the common myeloid progenitor (CMP) in humans. For eosinophil lineage commitment to occur, the myeloid progenitor (GMP or CMP) must express C/EBPα, C/EBPε, interferon regulatory factor 8 (IRF8), and PU.1. Expression of friend of GATA-1 (FOG-1) declines, allowing for increasing expression and activity of GATA TFs, which is necessary for EoP production. Following lineage commitment, eosinophil granule protein gene expression is markedly increased with the collaborative interaction between C/EBPε, PU.1, and GATA-1. To assist with the elevated granule protein synthesis in the EoP and eosinophil precursors, XBP1 expression is increased and promotes survival during the demanding maturation process. Expression of activator isoforms of C/EBPε peaks during eosinophil maturation and then declines during the final stages. Expression of ID2 increases during eosinophil maturation and enhances the rate of maturation.

The first stage in eosinophil development is commitment to the eosinophil lineage by a myeloid multipotent progenitor to generate an EoP (Figure 1). The EoP is identified *via* surface expression of CD34, interleukin 5 (IL-5) receptor alpha (IL-5Rα, a.k.a. CD125), and low levels of c-KIT (CD117) in murine bone marrow (1). In humans, EoPs are identified by surface expression of CD34, CD38, and CD125 (2). EoPs reside in small numbers primarily in the bone marrow (~0.05% of lineage-negative CD34^+^ cells), with even lower levels found in peripheral blood and in human umbilical cord blood (2). Targeting the EoP and the steps determining eosinophil lineage fate for treatment purposes is an attractive strategy, as it would prevent the production of mature eosinophils and all of their immune-activating contents; thus, delineating the factors that are essential for eosinophil lineage commitment will likely be clinically relevant.

### Eosinophil Lineage Instruction by GATA-1 and GATA-2

It is well established that myeloid progenitor expression of the TF GATA-1 is essential for eosinophil lineage commitment (6–9). The findings of these earlier studies were supported recently by global gene expression profiling of single murine multipotent progenitor cells revealing that the commitment to the eosinophil lineage segregated with *Gata1* expression (10). In addition, scRNA-seq of murine GMPs (Lin^−^CD34^+^c-KIT^+^CD16/32^hi^) revealed a rare GMP subset with eosinophil lineage potential and that maintained expression of *Gata1* (11).

Two nuclear factors, friend of GATA-1 (FOG-1; *Zfpm1*) and interferon regulatory factor 8 (IRF8; *Irf8* or *Icsbp*), have been shown to be important for regulating *Gata1* expression and/or function in myeloid progenitors and, consequently, to affect eosinophil production. FOG-1 is a transcriptional cofactor that facilitates binding of GATA factors to DNA and recruits chromatin remodeling complexes (12–14). FOG-1 is highly expressed by multipotent progenitors, antagonizes GATA-1 transcriptional activity, and must be downregulated to allow for eosinophil lineage commitment (15, 16). Loss of FOG-1 expression in mice is early embryonic lethal from severe anemia due to the requirement for FOG-1 for the formation of erythroid-lineage progenitors (17). FOG-1 deficiency in hematopoietic stem cells results in increased commitment along the myeloid lineages and aberrant expression of myeloid-related genes in megakaryocytic and erythroid cells (18), highlighting the role for FOG-1 in suppressing myeloid cell development. In contrast, loss of *Irf8* expression in mice resulted in reduced EoP (and eosinophil) frequency in the bone marrow and lower *Gata1* expression in the EoPs that were produced (19), suggesting that the TF IRF8 is critical for upregulating and/or maintaining GATA-1 expression in myeloid progenitors for eosinophil lineage commitment. Notably, murine GMPs with eosinophil lineage potential and that maintained *Gata1* expression also expressed intermediate levels of *Irf8* (11).

Murine EoPs express both GATA-1 and GATA-2, whereas GMPs express no GATA-1 and low to no level of GATA-2 (5, 20). Ectopic expression of GATA-2 in murine GMPs and human CD34^+^ hematopoietic progenitors was sufficient to instruct commitment to the eosinophil lineage (7, 20) and induce expression of GATA-1 (20). GATA-1 and GATA-2 have identical DNA sequence binding preferences, but their target genes and transcriptional responsibilities can be cell specific and/or overlapping, likely *via* a multitude of coregulators (e.g., FOG-1) (21). Targeted deletion of GATA-1 or GATA-2 has revealed that they control distinct biological processes that affect multiple hematopoietic lineages (21). Taken together, these studies emphasize the essential and instructive role for GATA TFs in eosinophil development; yet, targeting GATA-1 or GATA-2 therapeutically is likely to have significant and unacceptable effects on other hematopoietic lineages.

### C/EBPα Co-Expression with GATA-1 or GATA-2

In addition to expressing GATA-1 and GATA-2, EoPs express relatively high levels of the TF CCAAT/enhancer-binding protein alpha (C/EBPα) (20). C/EBPα is necessary for eosinophil development, as C/EBPα-deficient mice lack eosinophils (and neutrophils) (22). The level of C/EBPα expression is important for eosinophil- vs neutrophil-lineage commitment, as elevated expression of C/EBPα in GMPs due to an impaired protein degradation pathway results in increased neutrophil differentiation at the expense of eosinophils (23). In addition, the order of expression of GATA factors and C/EBPα is critical for eosinophil lineage commitment (8, 20, 24). Enforced expression of GATA-1 or GATA-2 in a C/EBPα-expressing progenitor results in eosinophil lineage commitment (20). In contrast, ectopic expression of GATA-2 prior to C/EBPα expression leads to basophil-lineage commitment (20). It is believed that C/EBPα is at least partially responsible for the downregulation of FOG-1 expression in myeloid progenitors promoting eosinophil development (15).

### C/EBPε Promotes Eosinophil Cell Fate

Multiple isoforms of the TF C/EBPε with distinct transcriptional functions (e.g., activators and repressors) are expressed during eosinophil maturation, and expression levels of the varying isoforms change with developmental stage (25, 26), reinforcing that ratios of TFs with combinatorial and even antagonistic activities are highlights of the eosinophil developmental program. Low levels of the activator C/EBPε isoforms are expressed in CD34^+^ hematopoietic progenitors, and all isoforms increase in expression during IL-5-mediated differentiation, with the repressor isoforms predominating during later stages of maturation (25). Mice deficient in C/EBPε fail to generate mature eosinophils or normal neutrophils (27), supporting a critical role for C/EBPε in a common upstream myeloid progenitor. Notably, ectopic expression of the activator isoforms of C/EBPε in umbilical cord blood CD34^+^ progenitors resulted in markedly increased commitment to the eosinophil lineage (25). In contrast, expression of the repressor isoforms decreased eosinophil cell fate, but not other myeloid lineages (25), suggesting that inducing expression of repressor isoforms in early myeloid progenitors may specifically inhibit eosinophil production. Expression of the four isoforms of C/EBPε results from differential splicing and alternative use of promoters (26, 28), but the critical transcriptional regulators that orchestrate the expression of the different isoforms is not known.

### Unclear Roles for PU.1

The TF PU.1 is a member of the ETS family of DNA-binding proteins with an essential function in both myeloid and lymphoid development (29, 30). Though the PU.1 expression level in myeloid progenitors has been shown to be important in regulating macrophage and neutrophil cell fates (3, 31), a definitive early role for PU.1 in eosinophil lineage commitment has not been defined. Gene expression analysis of PU.1-deficient fetal liver cells revealed expression of eosinophil peroxidase and major basic protein (*Prg2*), but little to no *Il5ra* (32), suggesting that PU.1 is not essential for eosinophil lineage commitment, but studies with a specific focus on the eosinophil lineage potential of hematopoietic cells deficient in PU.1 are needed.

### Summary of Eosinophil Lineage Commitment

In summary, eosinophil lineage commitment occurs in a myeloid multipotent progenitor that expresses C/EBPα, C/EBPε, and IRF8 followed by concomitant declining FOG-1 expression and increasing GATA-1 and GATA-2 expression (Figure 1). This hierarchical combination of TFs has been shown to be necessary for eosinophil lineage commitment.

## Eosinophil Maturation

Human eosinophils have characteristic morphologic features, including a bilobed nucleus and cytoplasmic granules filled with cationic proteins that are packaged in a specific manner (Figure 1). Eosinophils are terminally differentiated and do not proliferate once they leave the bone marrow. We noted that mature eosinophils share expression of 60 TFs with EoPs and express an additional 35 TFs that EoPs do not (5), suggesting that it requires a greater number of TFs to produce a more complex and differentiated cell. Identifying the critical TFs for specific eosinophil functional responses will provide potential new therapeutic targets.

### PU.1 Priming for Transcription

Recent studies in macrophages have revealed a collaborative interaction between PU.1 and other lineage-determining TFs, such as C/EBPα, to open chromatin and “prime” genes for transcription (33, 34). Consistent with this role as a “pioneer” TF, PU.1 has been shown to cooperatively regulate the expression of eosinophil granule protein genes (35–37), including *PRG2* (major basic protein) and *RNS2* (eosinophil-derived neurotoxin), highlighting an important role for PU.1 in eosinophil maturation. Future studies are needed to determine how the distribution of PU.1 across the genome differs between granulocytes (eosinophils, neutrophils, basophils, and mast cells) and what partnerships are critical for terminal differentiation of the distinct cell types.

### C/EBPε Interaction with PU.1

One of the PU.1 collaborators in regulating gene expression during eosinophil maturation is the TF C/EBPε. The peripheral blood and bone marrow of adult mice deficient in C/EBPε have a pronounced increase in immature myeloid precursors, indicating a blockade in terminal granulocyte differentiation in the absence of C/EBPε (27). In addition, ectopic expression of C/EBPε in CD34^+^ hematopoietic progenitors increased the rate of eosinophil maturation (25). C/EBPε is important for the expression of secondary granules in both neutrophils and eosinophils (36, 37), and C/EBPε deficiency results in impaired functional responses for neutrophils (27). Individuals with mutations that abolish C/EBPε expression produce abnormal neutrophils and eosinophils that lack specific granules; thus, these individuals suffer from early and frequent bacterial infections (26, 38, 39), providing clinically relevant support for a critical role for C/EBPε in terminal differentiation of granulocytes. Interestingly, peripheral blood eosinophils predominantly express one of the repressor isoforms of C/EBPε (36), suggesting that C/EBPε’s repressive activity is more important during late-stage eosinophil maturation.

### XBP1 Is Required for EoP Survival

Murine EoPs have been shown to contain nascent granules (1, 5) and express granule protein mRNAs at a higher level than mature eosinophils (5); thus, early EoP differentiation likely represents a developmentally restricted period during eosinophilopoiesis when protein production and endoplasmic reticulum (ER) demand peaks. XBP1 (*Xbp1*) is a TF that is involved in the unfolded protein response triggered by ER stress (40). In response to ER stress, *Xbp1* mRNA is spliced by the endoribonuclease IRE1α followed by translation of the active TF XBP1. Accumulation of the spliced *Xbp1* mRNA was higher in GMPs and EoPs than eosinophil precursors, and no spliced *Xbp1* mRNA was noted in mature eosinophils, which is consistent with activation of the ER stress pathway during high protein synthetic demands through eosinophil maturation (41). Notably, loss of *Xbp1* expression in hematopoietic cells resulted in a compete loss of mature eosinophils (41). EoPs were present in the bone marrow but at a lower frequency in *Xbp1*-deficient than *Xbp1*-sufficient mice, likely due to poor survival (41); thus, *Xbp1* is essential for eosinophil maturation but not lineage commitment.

### ID2 Enhances Terminal Differentiation

Inhibitor of DNA-binding (ID) proteins is a family of negative transcriptional regulators that heterodimerizes with basic helix-loop-helix TFs and prevents binding to the DNA (42). Expression of ID2 was upregulated during eosinophil maturation, and ectopic expression of ID2 in human CD34^+^ hematopoietic progenitors resulted in increased mature eosinophils, with no change in frequency of the earlier precursors (43), suggesting that ID2 enhances terminal differentiation. In contrast, expression of ID1 declines during eosinophil maturation and inhibits terminal differentiation (43).

## Eosinophil Function

In addition to orchestrating eosinophil production, TFs also participate in eosinophil functional responses and survival. Glucocorticoids are the first-line therapy for eosinophil-associated disorders, such as allergy, asthma, eosinophilic gastrointestinal disorders and hypereosinophilic syndrome (44, 45); yet, there are a subset of individuals with severe asthma with eosinophilia despite high doses of glucocorticoids (46–48) and patients with hypereosinophilic syndrome often become glucocorticoid refractory (49, 50). The TF NFIL3 has recently been shown to be induced by IL-5 stimulation in eosinophils and to protect against glucocorticoid-induced apoptosis (51), suggesting that targeting NFIL3 in patients may restore glucocorticoid sensitivity. STAT6 is another TF that has been shown to regulate eosinophil functional responses, specifically in experimental asthma. Sensitized mice with STAT6-deficient eosinophils were protected against mucus overproduction and airway hyperresponsiveness following allergen challenge (52), highlighting an important role for STAT6 signaling in eosinophils in allergic asthma. Yet, eosinophil-intrinsic STAT6 was not required for eosinophil recruitment into tissues in response to parasitic infection (53), highlighting the need for further investigations to delineate the impact of environmental signals on gene regulatory programs. Together, these studies suggest that targeting TFs in specific clinical settings may impact eosinophil function and survival.

## Conclusion and Future Directions

As there have been no described TFs that are specific to the eosinophil lineage, targeting eosinophil production currently has been achieved primarily *via* indirect means. A wealth of evidence support a critical role for the cytokine IL-5 in mediating disease-associated eosinophilia, and neutralizing IL-5 indirectly suppresses eosinophil maturation (54). IL-5 is produced by type 2 helper T (Th2) cells and the TF GATA-3 has been shown to control expression of IL-5 in Th2 cells (55). In addition, group 2 innate lymphoid cells (ILC2s) produce large amounts of IL-5 upon activation by epithelial-derived cytokines (56, 57) and GATA-3 is essential for ILC2 development (58); thus, GATA-3 is an attractive therapeutic target to prevent IL-5 expression. Notably, treatment with a DNA enzyme that cleaved *GATA3* mRNA resulted in reduced airway eosinophilia and plasma levels of IL-5 in individuals with asthma (59, 60), highlighting the feasibility of targeting TFs in patients with eosinophil disorders. With emerging technology and public databases of information available to investigators around the world, the future for research in eosinophil development is bright. Many new questions have arisen as our knowledge expands. Recently, a new regulatory eosinophil subset has been described in the murine lung and with a transcriptome that differed from that of inflammatory eosinophils (61). In addition, thymus-resident eosinophils have a distinct phenotype from other tissue-resident eosinophils (62). Together, these studies indicate that extrinsic signals from the local environment likely affect gene expression *via* changes in the regulatory program or that these eosinophil subsets are produced *via* a differential developmental program. Understanding how specific signals direct distinct patterns of gene expression required for the specialized functions of tissue-resident eosinophils will likely lead to new targets for therapeutic intervention.

## Author Contributions

The author confirms being the sole contributor of this work and approved it for publication.

## Conflict of Interest Statement

The author declares that the research was conducted in the absence of any commercial or financial relationships that could be construed as a potential conflict of interest.

## References

[1] Iwasaki H Mizuno S Mayfield R Shigematsu H Arinobu Y Seed B Identification of eosinophil lineage-committed progenitors in the murine bone marrow. J Exp Med (2005) 201(12):1891–7. 10.1084/jem.20050548 15955840 PMC2212039

[2] Mori Y Iwasaki H Kohno K Yoshimoto G Kikushige Y Okeda A Identification of the human eosinophil lineage-committed progenitor: revision of phenotypic definition of the human common myeloid progenitor. J Exp Med (2009) 206(1):183–93. 10.1084/jem.20081756 19114669 PMC2626675

[3] Dahl R Walsh JC Lancki D Laslo P Iyer SR Singh H Regulation of macrophage and neutrophil cell fates by the PU.1:C/EBPalpha ratio and granulocyte colony-stimulating factor. Nat Immunol (2003) 4(10):1029–36. 10.1038/ni973 12958595

[4] Walsh JC DeKoter RP Lee HJ Smith ED Lancki DW Gurish MF Cooperative and antagonistic interplay between PU.1 and GATA-2 in the specification of myeloid cell fates. Immunity (2002) 17(5):665–76. 10.1016/S1074-7613(02)00452-1 12433372

[5] Bouffi C Kartashov AV Schollaert KL Chen X Bacon WC Weirauch MT Transcription factor repertoire of homeostatic eosinophilopoiesis. J Immunol (2015) 195(6):2683–95. 10.4049/jimmunol.1500510 26268651 PMC4561201

[6] Kulessa H Frampton J Graf T. GATA-1 reprograms avian myelomonocytic cell lines into eosinophils, thromboblasts, and erythroblasts. Genes Dev (1995) 9(10):1250–62. 10.1101/gad.9.10.1250 7758949

[7] Hirasawa R Shimizu R Takahashi S Osawa M Takayanagi S Kato Y Essential and instructive roles of GATA factors in eosinophil development. J Exp Med (2002) 195(11):1379–86. 10.1084/jem.20020170 12045236 PMC2193540

[8] McNagny K Graf T Making eosinophils through subtle shifts in transcription factor expression. J Exp Med (2002) 195(11):f43–7. 10.1084/jem.20020636 12045250 PMC2193544

[9] Yu C Cantor AB Yang H Browne C Wells RA Fujiwara Y Targeted deletion of a high-affinity GATA-binding site in the GATA-1 promoter leads to selective loss of the eosinophil lineage in vivo. J Exp Med (2002) 195(11):1387–95. 10.1084/jem.20020656 12045237 PMC2193547

[10] Drissen R Buza-Vidas N Woll P Thongjuea S Gambardella A Giustacchini A Distinct myeloid progenitor-differentiation pathways identified through single-cell RNA sequencing. Nat Immunol (2016) 17(6):666–76. 10.1038/ni.3412 27043410 PMC4972405

[11] Olsson A Venkatasubramanian M Chaudhri VK Aronow BJ Salomonis N Singh H Single-cell analysis of mixed-lineage states leading to a binary cell fate choice. Nature (2016) 537(7622):698–702. 10.1038/nature19348 27580035 PMC5161694

[12] Tsang AP Visvader JE Turner CA Fujiwara Y Yu C Weiss MJ FOG, a multitype zinc finger protein, acts as a cofactor for transcription factor GATA-1 in erythroid and megakaryocytic differentiation. Cell (1997) 90(1):109–19. 10.1016/S0092-8674(00)80318-9 9230307

[13] Fox AH Liew C Holmes M Kowalski K Mackay J Crossley M. Transcriptional cofactors of the FOG family interact with GATA proteins by means of multiple zinc fingers. EMBO J (1999) 18(10):2812–22. 10.1093/emboj/18.10.2812 10329627 PMC1171362

[14] Pal S Cantor AB Johnson KD Moran TB Boyer ME Orkin SH Coregulator-dependent facilitation of chromatin occupancy by GATA-1. Proc Natl Acad Sci U S A (2004) 101(4):980–5. 10.1073/pnas.0307612100 14715908 PMC327128

[15] Querfurth E Schuster M Kulessa H Crispino JD Doderlein G Orkin SH Antagonism between C/EBPbeta and FOG in eosinophil lineage commitment of multipotent hematopoietic progenitors. Genes Dev (2000) 14(19):2515–25. 10.1101/gad.177200 11018018 PMC316981

[16] Du Roure C Versavel A Doll T Cao C Pillonel V Matthias G Hematopoietic overexpression of FOG1 does not affect B-cells but reduces the number of circulating eosinophils. PLoS One (2014) 9(4):e92836. 10.1371/journal.pone.0092836 24747299 PMC3991581

[17] Tsang AP Fujiwara Y Hom DB Orkin SH. Failure of megakaryopoiesis and arrested erythropoiesis in mice lacking the GATA-1 transcriptional cofactor FOG. Genes Dev (1998) 12(8):1176–88. 10.1101/gad.12.8.1176 9553047 PMC316724

[18] Mancini E Sanjuan-Pla A Luciani L Moore S Grover A Zay A FOG-1 and GATA-1 act sequentially to specify definitive megakaryocytic and erythroid progenitors. EMBO J (2012) 31(2):351–65. 10.1038/emboj.2011.390 22068055 PMC3261555

[19] Milanovic M Terszowski G Struck D Liesenfeld O Carstanjen D. IFN consensus sequence binding protein (Icsbp) is critical for eosinophil development. J Immunol (2008) 181(7):5045–53. 10.4049/jimmunol.181.7.5045 18802108

[20] Iwasaki H Mizuno S Arinobu Y Ozawa H Mori Y Shigematsu H The order of expression of transcription factors directs hierarchical specification of hematopoietic lineages. Genes Dev (2006) 20(21):3010–21. 10.1101/gad.1493506 17079688 PMC1620021

[21] Katsumura KR Bresnick EH Group GFM. The GATA factor revolution in hematology. Blood (2017) 129(15):2092–102. 10.1182/blood-2016-09-687871 28179282 PMC5391619

[22] Zhang DE Zhang P Wang ND Hetherington CJ Darlington GJ Tenen DG. Absence of granulocyte colony-stimulating factor signaling and neutrophil development in CCAAT enhancer binding protein alpha-deficient mice. Proc Natl Acad Sci U S A (1997) 94(2):569–74. 10.1073/pnas.94.2.569 9012825 PMC19554

[23] Satoh T Kidoya H Naito H Yamamoto M Takemura N Nakagawa K Critical role of Trib1 in differentiation of tissue-resident M2-like macrophages. Nature (2013) 495(7442):524–8. 10.1038/nature11930 23515163

[24] Nerlov C McNagny KM Doderlein G Kowenz-Leutz E Graf T. Distinct C/EBP functions are required for eosinophil lineage commitment and maturation. Genes Dev (1998) 12(15):2413–23. 10.1101/gad.12.15.2413 9694805 PMC317049

[25] Bedi R Du J Sharma AK Gomes I Ackerman SJ. Human C/EBP-epsilon activator and repressor isoforms differentially reprogram myeloid lineage commitment and differentiation. Blood (2009) 113(2):317–27. 10.1182/blood-2008-02-139741 18832658 PMC2615649

[26] Lekstrom-Himes JA. The role of C/EBP(epsilon) in the terminal stages of granulocyte differentiation. Stem Cells (2001) 19(2):125–33. 10.1634/stemcells.19-2-125 11239167

[27] Yamanaka R Barlow C Lekstrom-Himes J Castilla LH Liu PP Eckhaus M Impaired granulopoiesis, myelodysplasia, and early lethality in CCAAT/enhancer binding protein epsilon-deficient mice. Proc Natl Acad Sci U S A (1997) 94(24):13187–92. 10.1073/pnas.94.24.13187 9371821 PMC24284

[28] Yamanaka R Kim GD Radomska HS Lekstrom-Himes J Smith LT Antonson P CCAAT/enhancer binding protein epsilon is preferentially up-regulated during granulocytic differentiation and its functional versatility is determined by alternative use of promoters and differential splicing. Proc Natl Acad Sci U S A (1997) 94(12):6462–7. 10.1073/pnas.94.12.6462 9177240 PMC21072

[29] McKercher SR Torbett BE Anderson KL Henkel GW Vestal DJ Baribault H Targeted disruption of the PU.1 gene results in multiple hematopoietic abnormalities. EMBO J (1996) 15(20):5647–58. 8896458 PMC452309

[30] Scott EW Simon MC Anastasi J Singh H. Requirement of transcription factor PU.1 in the development of multiple hematopoietic lineages. Science (1994) 265(5178):1573–7. 10.1126/science.8079170 8079170

[31] Dahl R Simon MC. The importance of PU.1 concentration in hematopoietic lineage commitment and maturation. Blood Cells Mol Dis (2003) 31(2):229–33. 10.1016/S1079-9796(03)00152-9 12972030

[32] Lee J Rosenberg HF Eosinophils in Health and Disease. 1st ed London, Waltham, MA: Elsevier/Academic Press (2013). xxiii, 654 p.

[33] Heinz S Glass CK. Roles of lineage-determining transcription factors in establishing open chromatin: lessons from high-throughput studies. Curr Top Microbiol Immunol (2012) 356:1–15. 10.1007/82_2011_142 21744305

[34] Heinz S Romanoski CE Benner C Glass CK. The selection and function of cell type-specific enhancers. Nat Rev Mol Cell Biol (2015) 16(3):144–54. 10.1038/nrm3949 25650801 PMC4517609

[35] van Dijk TB Caldenhoven E Raaijmakers JA Lammers JW Koenderman L de Groot RP. The role of transcription factor PU.1 in the activity of the intronic enhancer of the eosinophil-derived neurotoxin (RNS2) gene. Blood (1998) 91(6):2126–32. 9490699

[36] Du J Stankiewicz MJ Liu Y Xi Q Schmitz JE Lekstrom-Himes JA Novel combinatorial interactions of GATA-1, PU.1, and C/EBPepsilon isoforms regulate transcription of the gene encoding eosinophil granule major basic protein. J Biol Chem (2002) 277(45):43481–94. 10.1074/jbc.M204777200 12202480

[37] Gombart AF Kwok SH Anderson KL Yamaguchi Y Torbett BE Koeffler HP. Regulation of neutrophil and eosinophil secondary granule gene expression by transcription factors C/EBP epsilon and PU.1. Blood (2003) 101(8):3265–73. 10.1182/blood-2002-04-1039 12515729

[38] Gombart AF Shiohara M Kwok SH Agematsu K Komiyama A Koeffler HP Neutrophil-specific granule deficiency: homozygous recessive inheritance of a frameshift mutation in the gene encoding transcription factor CCAAT/enhancer binding protein–epsilon. Blood (2001) 97(9):2561–7. 10.1182/blood.V97.9.2561 11313242

[39] Rosenberg HF Gallin JI. Neutrophil-specific granule deficiency includes eosinophils. Blood (1993) 82(1):268–73. 8324226

[40] Calfon M Zeng H Urano F Till JH Hubbard SR Harding HP IRE1 couples endoplasmic reticulum load to secretory capacity by processing the XBP-1 mRNA. Nature (2002) 415(6867):92–6. 10.1038/415092a 11780124

[41] Bettigole SE Lis R Adoro S Lee AH Spencer LA Weller PF The transcription factor XBP1 is selectively required for eosinophil differentiation. Nat Immunol (2015) 16(8):829–37. 10.1038/ni.3225 26147683 PMC4577297

[42] Kee BL. E and ID proteins branch out. Nat Rev Immunol (2009) 9(3):175–84. 10.1038/nri2507 19240756

[43] Buitenhuis M van Deutekom HW Verhagen LP Castor A Jacobsen SE Lammers JW Differential regulation of granulopoiesis by the basic helix-loop-helix transcriptional inhibitors Id1 and Id2. Blood (2005) 105(11):4272–81. 10.1182/blood-2004-12-4883 15701714

[44] Klion AD. Eosinophilia: a pragmatic approach to diagnosis and treatment. Hematology Am Soc Hematol Educ Program (2015) 2015:92–7. 10.1182/asheducation-2015.1.92 26637706

[45] Uppal V Kreiger P Kutsch E. Eosinophilic gastroenteritis and colitis: a comprehensive review. Clin Rev Allergy Immunol (2016) 50(2):175–88. 10.1007/s12016-015-8489-4 26054822

[46] Nair P Pizzichini MM Kjarsgaard M Inman MD Efthimiadis A Pizzichini E Mepolizumab for prednisone-dependent asthma with sputum eosinophilia. N Engl J Med (2009) 360(10):985–93. 10.1056/NEJMoa0805435 19264687

[47] Pavord ID Korn S Howarth P Bleecker ER Buhl R Keene ON Mepolizumab for severe eosinophilic asthma (DREAM): a multicentre, double-blind, placebo-controlled trial. Lancet (2012) 380(9842):651–9. 10.1016/S0140-6736(12)60988-X 22901886

[48] Pavord ID Haldar P Bradding P Wardlaw AJ Mepolizumab in refractory eosinophilic asthma. Thorax (2010) 65(4):370 10.1136/thx.2009.122697 20388767

[49] Debierre-Grockiego F Leduc I Prin L Gouilleux-Gruart V. Dexamethasone inhibits apoptosis of eosinophils isolated from hypereosinophilic patients. Immunobiology (2001) 204(4):517–23. 10.1078/0171-2985-00060 11776405

[50] Ogbogu PU Bochner BS Butterfield JH Gleich GJ Huss-Marp J Kahn JE Hypereosinophilic syndrome: a multicenter, retrospective analysis of clinical characteristics and response to therapy. J Allergy Clin Immunol (2009) 124(6):1319–25.e3. 10.1016/j.jaci.2009.09.022 19910029 PMC2829669

[51] Pazdrak K Moon Y Straub C Stafford S Kurosky A. Eosinophil resistance to glucocorticoid-induced apoptosis is mediated by the transcription factor NFIL3. Apoptosis (2016) 21(4):421–31. 10.1007/s10495-016-1226-5 26880402 PMC4769953

[52] Stokes K LaMarche NM Islam N Wood A Huang W August A. Cutting edge: STAT6 signaling in eosinophils is necessary for development of allergic airway inflammation. J Immunol (2015) 194(6):2477–81. 10.4049/jimmunol.1402096 25681342 PMC4470169

[53] Voehringer D van Rooijen N Locksley RM. Eosinophils develop in distinct stages and are recruited to peripheral sites by alternatively activated macrophages. J Leukoc Biol (2007) 81(6):1434–44. 10.1189/jlb.1106686 17339609

[54] Molfino NA Gossage D Kolbeck R Parker JM Geba GP. Molecular and clinical rationale for therapeutic targeting of interleukin-5 and its receptor. Clin Exp Allergy (2012) 42(5):712–37. 10.1111/j.1365-2222.2011.03854.x 22092535

[55] Ray A Cohn L Th2 cells and GATA-3 in asthma: new insights into the regulation of airway inflammation. J Clin Invest (1999) 104(8):985–93. 10.1172/JCI8204 10525032 PMC408864

[56] Neill DR Wong SH Bellosi A Flynn RJ Daly M Langford TK Nuocytes represent a new innate effector leukocyte that mediates type-2 immunity. Nature (2010) 464(7293):1367–70. 10.1038/nature08900 20200518 PMC2862165

[57] Saenz SA Siracusa MC Perrigoue JG Spencer SP Urban JF Jr Tocker JE IL25 elicits a multipotent progenitor cell population that promotes T(H)2 cytokine responses. Nature (2010) 464(7293):1362–6. 10.1038/nature08901 20200520 PMC2861732

[58] Tindemans I Serafini N Di Santo JP Hendriks RW. GATA-3 function in innate and adaptive immunity. Immunity (2014) 41(2):191–206. 10.1016/j.immuni.2014.06.006 25148023

[59] Homburg U Renz H Timmer W Hohlfeld JM Seitz F Luer K Safety and tolerability of a novel inhaled GATA3 mRNA targeting DNAzyme in patients with TH2-driven asthma. J Allergy Clin Immunol (2015) 136(3):797–800. 10.1016/j.jaci.2015.02.018 25842286

[60] Krug N Hohlfeld JM Kirsten AM Kornmann O Beeh KM Kappeler D Allergen-induced asthmatic responses modified by a GATA3-specific DNAzyme. N Engl J Med (2015) 372(21):1987–95. 10.1056/NEJMoa1411776 25981191

[61] Mesnil C Raulier S Paulissen G Xiao X Birrell MA Pirottin D Lung-resident eosinophils represent a distinct regulatory eosinophil subset. J Clin Invest (2016) 126(9):3279–95. 10.1172/JCI85664 27548519 PMC5004964

[62] Becher B Schlitzer A Chen J Mair F Sumatoh HR Teng KW High-dimensional analysis of the murine myeloid cell system. Nat Immunol (2014) 15(12):1181–9. 10.1038/ni.3006 25306126

